# Supplementary figures and images for: Diverse Viruses in Deep-Sea Hydrothermal Vent Fluids Have Restricted Dispersal across Ocean Basins
Source: mSystems. 2021 Jun 22;6(3):e00068-21. doi: 10.1128/mSystems.00068-21 (PMC8269205; doi:10.1128/mSystems.00068-21)

A.

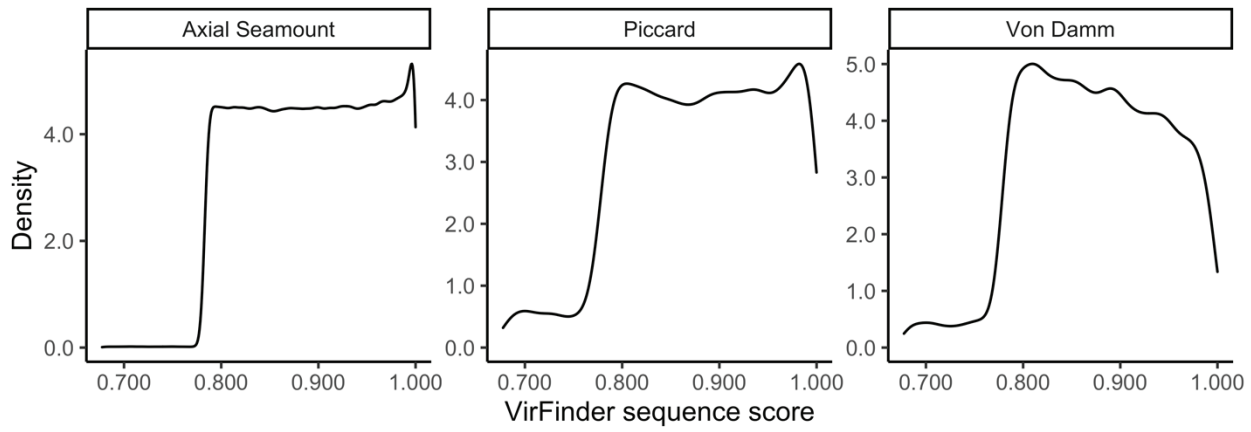

B.

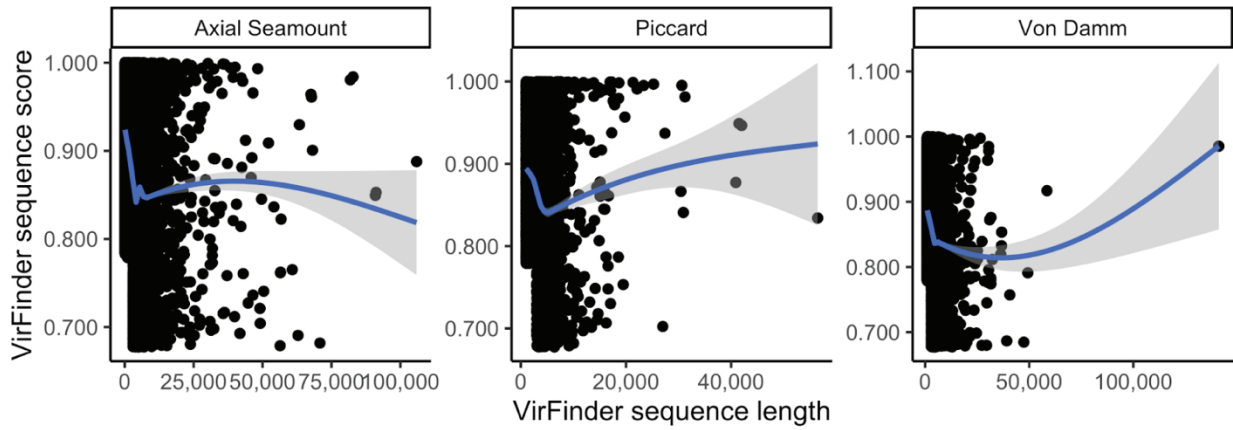

Supplement: FIG S1 [file msystems.00068-21-sf001.pdf]

A.

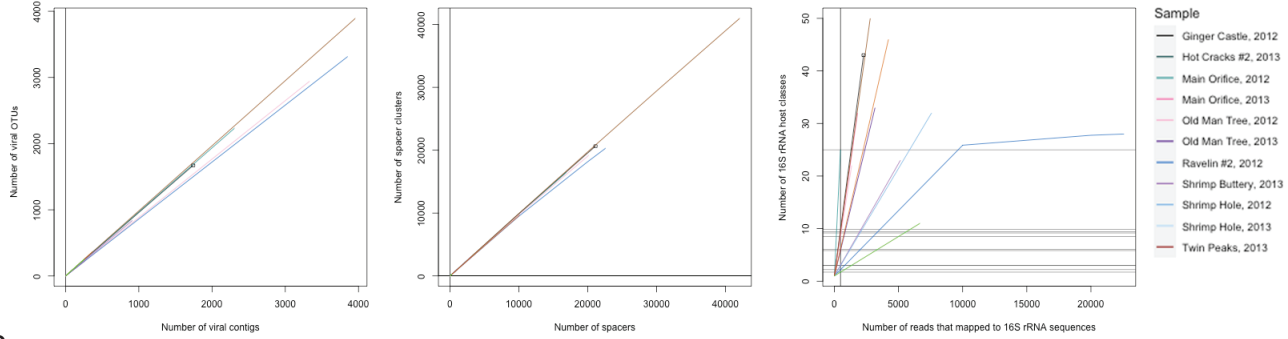

B.

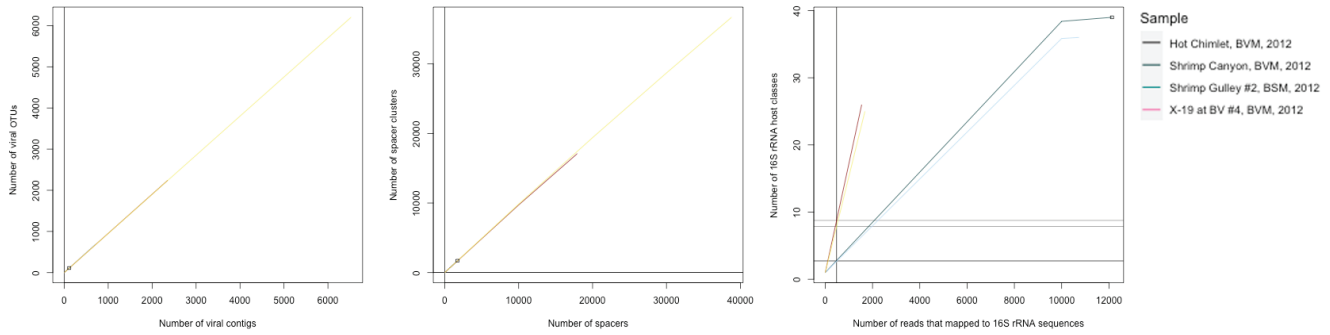

C.

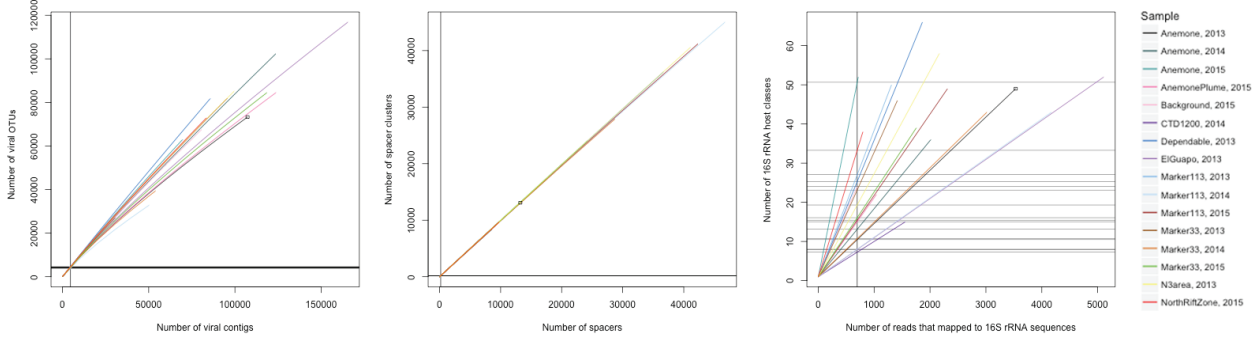

Supplement: FIG S2 [file msystems.00068-21-sf002.pdf]

A.

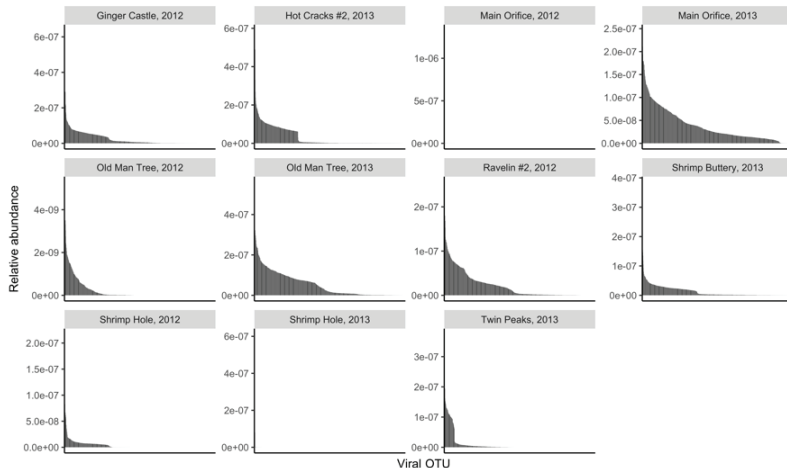

B.

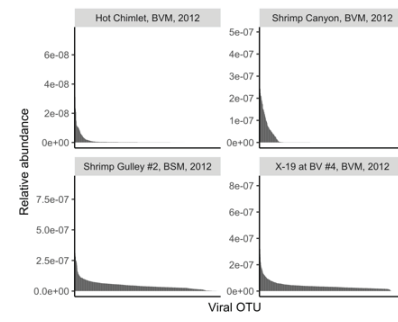

C.

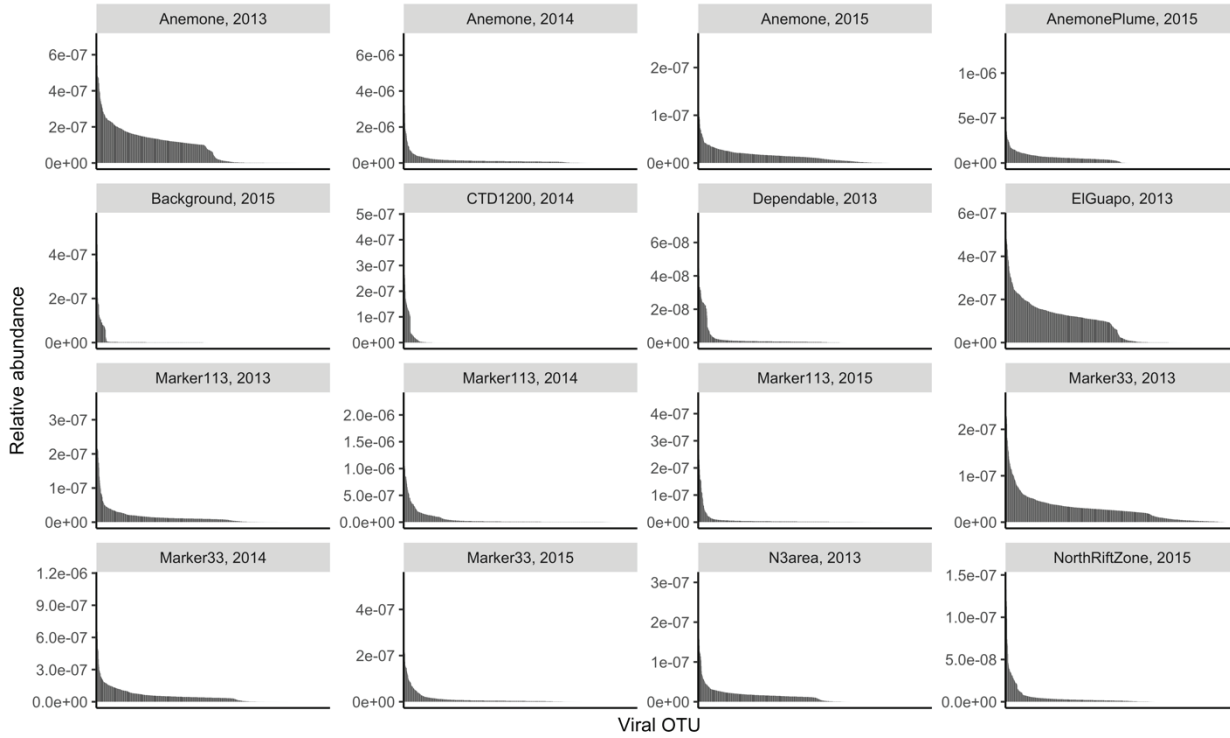

Supplement: FIG S3 [file msystems.00068-21-sf003.pdf]

A.

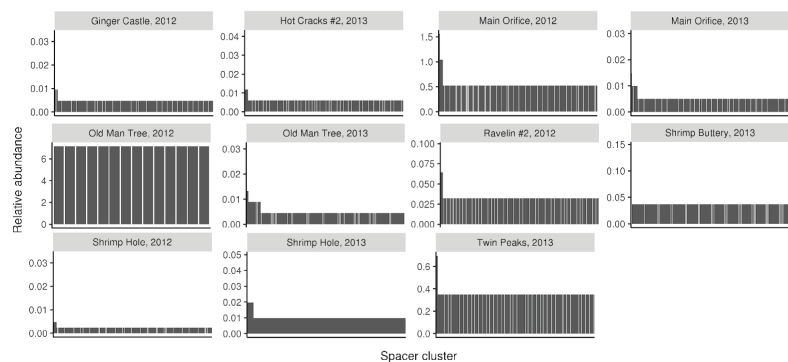

B.

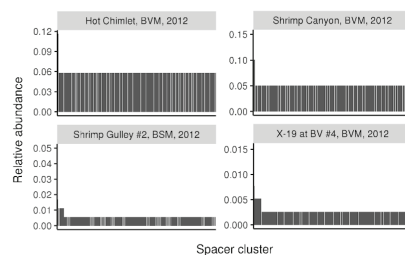

C.

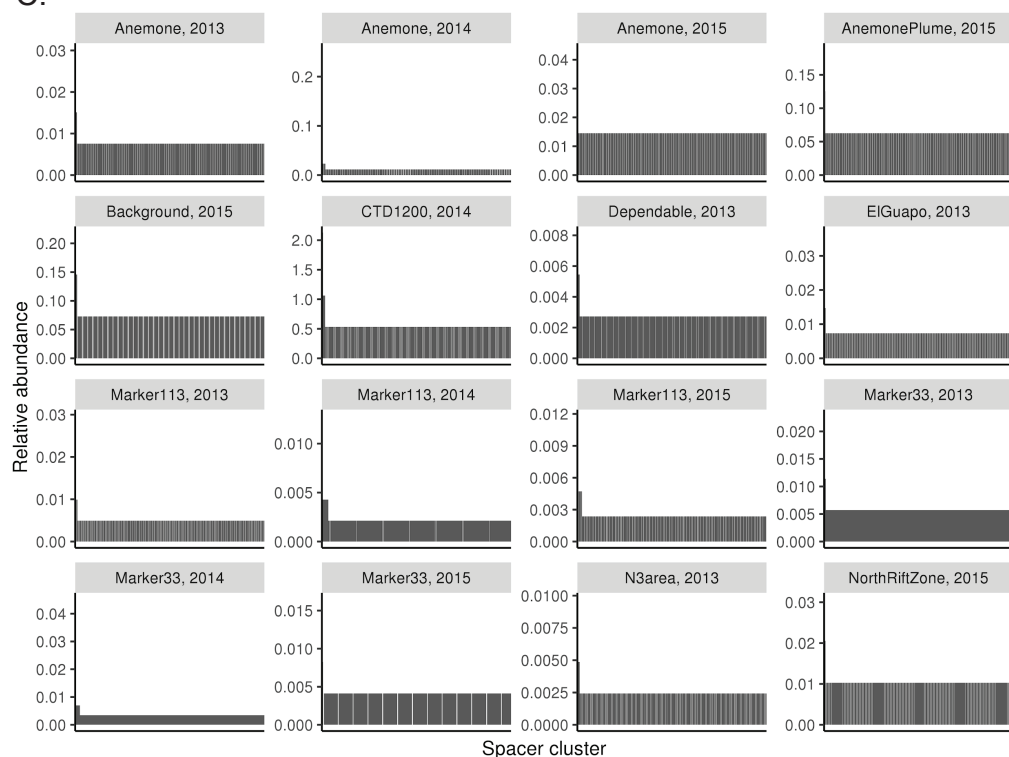

Supplement: FIG S4 [file msystems.00068-21-sf004.pdf]
