# Supplementary material for: Diverse Viruses in Deep-Sea Hydrothermal Vent Fluids Have Restricted Dispersal across Ocean Basins
Source: mSystems. 2021 Jun 22;6(3):e00068-21. doi: 10.1128/mSystems.00068-21 (PMC8269205; doi:10.1128/mSystems.00068-21)
Supplement: TEXT S1 [file msystems.00068-21-t0001.docx]

**SUPPLEMENTARY MATERIAL**

*Viruses and prophages in MAGS and viral and prophage genes*

In order to identify viral genes and putative auxiliary metabolic genes (AMGs), VirSorter (1) v1.0.3 was used to extract putative viral and prophage sequences from metagenomic assemblies and from individual MAGs. In the case of identifying putative auxiliary metabolic genes, we chose to maximize accuracy rather than recovery. Therefore, we used VirSorter for prophage and AMG identification because VirSorter relies on known viral hallmark genes for viral identification and thus is a more conservative tool for positive viral identification. Category one, category two, category four (prophage), and category five (prophage) VirSorter sequences were used in analyses. Putative viral and prophage sequences from all assembled contigs were annotated via Prokka (2) v1.14 with the taxonomic identifier set to “bacteria” as well as to “viruses.” We assigned categories to the ORFs that had been identified with the “bacteria” taxonomic identifier using the Clusters of Orthologous Groups of proteins (COG) database (3, 4). We conducted a second round of annotation for ORFs identified by Prokka using the “viruses” taxonomic identifier using VirSorter; we classified these ORFs as having viral function if they were annotated with terms that included “capsid,” “tail,” ”spike,” “terminase large subunit,” “portal,” and “coat.”

Of the 74 Mid-Cayman Rise MAGs, 28 (38%) had at least one putative prophage sequence counting VirSorter categories one, two, four, or five, and 6 (8%) had a putative prophage counting only VirSorter categories four and five ([Supplementary Table 4](https://drive.google.com/open?id=1_gUOtzMeCBoUXuf50JUPrUglD6H2ahVW)). Of the 98 Axial Seamount MAGs, 41 (42%) had at least one putative viral or prophage sequence counting VirSorter categories one, two, four, or five, and 6 (6%) had a putative prophage sequence counting VirSorter categories four and five ([Supplementary Table 4](https://drive.google.com/open?id=1_gUOtzMeCBoUXuf50JUPrUglD6H2ahVW)). Given the uncertainties associated with binning viral contigs into MAGs, the true number of prophage in hydrothermal vent microbial genomes is likely between these endmember values. VirSorter has not been verified in hydrothermal systems; however, it is commonly used in other non-surface ocean systems (5–7).

To determine whether viruses and prophage in these samples carried auxiliary metabolic genes (AMGs), we annotated and characterized viral and prophage gene content in the viral sequences recovered from the metagenomes. The vast majority of genes within the putative viral and prophage sequences were annotated as belonging to the COG categories replication and repair; nucleotide metabolism and transport; and post-translational modification, protein turnover, chaperone functions. Many genes were also annotated as belonging to the cell wall/membrane/envelope biogenesis category. Within the Mid-Cayman Rise, a total of 106 ORFs within putative viral or prophage sequences identified in 10 different metagenomes fell within the broad COG category of “metabolism” (Supplementary Fig. 6). This included genes categorized as energy production and conversion, amino acid transport and metabolism, nucleotide transport and metabolism, carbohydrate transport and metabolism, coenzyme transport and metabolism, lipid transport and metabolism, and inorganic ion transport and metabolism. Samples taken from X-19 and Shrimp Hole (2012) had a high number of metabolism genes (30 and 28, respectively) on putative viral or prophage sequences compared to the other Mid-Cayman Rise samples. A higher proportion of genes on putative viral or prophage sequences were related to metabolism at the Mid-Cayman Rise than Axial Seamount (Supplementary Fig. 6). At Axial Seamount, we identified a total of 185 ORFs on putative viral or prophage sequences from 14 different metagenomes that fell within the broad COG category of “metabolism.” However, it is important to note that none of the observed metabolism genes were surrounded by confirmed virus genes (i.e., known viral ORFs both upstream and downstream of the metabolic ORF), and therefore we cannot rule out the possibility that some of these genes were microbial in origin.

*Auxiliary metabolic genes and the role of virus-driven horizontal gene transfer in these systems*

Previous work has suggested that viruses in hydrothermal systems have the genomic capacity to alter their hosts’ microbial metabolism through harboring auxiliary metabolic genes (AMGs) (8–10). We found a higher percentage of microbial genomes with putative viral sequences (40%) than has been previously reported for single-cell genomes (SAGs, 10%) in diffuse flow hydrothermal fluids (11). The high abundance of prophage identified here is consistent with previous work documenting a high incidence of prophage in diffuse flow systems compared to deep seawater (12).

The ORFs on the viral contigs identified in these hydrothermal vent metagenomes encoded a wide range of functions, some of which may function as auxiliary metabolic genes. Many viral contigs contained genes related to cell wall or membrane proteins. The function of these genes is unknown; it is possible that these genes are involved in the synthesis of membranes surrounding viral capsids. Genes related to outer membrane proteins and protein glycosylation are commonly observed as variable genes within microbial pangenomes (13–19), possibly as a means to vary membrane proteins to evade viral infection. Viruses often act as a source of genes for horizontal gene transfer via transduction, and it is possible that the introduction of these novel genes may enable some microbial strains to avoid infection by other viruses. We also observed several genes with functions related to energy metabolism, inorganic ion transport, and signal transduction. Previous work has found that genes related to inorganic ion transport are differentially distributed in the *Sulfurovum* pangenome according to nutrient availability (20) and further work on viral sequences across vent sites is needed to determine whether these genes are carried by viruses to benefit their hosts. Moreover, work on viruses in hydrothermal plumes (8) and in diffuse flow vent fluid (9) has indicated that viruses in these systems encode energy-metabolizing AMGs, potentially to supplement the host’s ability to generate sufficient energy for cellular processes during the course of infection. The abundance and diversity of metabolic genes observed on these contigs provide further evidence that viral-encoded AMGs are widespread and diverse within hydrothermal vent ecosystems. These genes also have the potential to be horizontally transferred via viral transduction. However, our analyses of viral biogeography in hydrothermal vents has revealed virus-host interactions to have limited distributions because viruses are spatially restricted and highly host-specific. Thus, the potential for viruses to act as mechanisms for horizontal gene transfer between spatially and phylogenetically distant hosts is likely limited.

**REFERENCES**

1. Roux S, Enault F, Hurwitz BL, Sullivan MB. 2015. VirSorter: mining viral signal from microbial genomic data. PeerJ 3:e985.

2. Seemann T. 2014. Prokka: rapid prokaryotic genome annotation. Bioinformatics 30:2068–2069.

3. Tatusov RL, Koonin E V., Lipman DJ. 1997. A genomic perspective on protein families. Science (80- ) 278:631–637.

4. Galperin MY, Wolf YI, Makarova KS, Alvarez RV, Landsman D, Koonin E V. 2021. COG database update: Focus on microbial diversity, model organisms, and widespread pathogens. Nucleic Acids Res 49:D274–D281.

5. Emerson JB, Roux S, Brum JR, Bolduc B, Woodcroft BJ, Jang H Bin, Singleton CM, Solden LM, Naas AE, Boyd JA, Hodgkins SB, Wilson RM, Trubl G, Li C, Frolking S, Pope PB, Wrighton KC, Crill PM, Chanton JP, Saleska SR, Tyson GW, Rich VI, Sullivan MB. 2018. Host-linked soil viral ecology along a permafrost thaw gradient. Nat Microbiol 1.

6. Trubl G, Jang H Bin, Roux S, Emerson JB, Solonenko N, Vik DR, Solden L, Ellenbogen J, Runyon AT, Bolduc B, Woodcroft BJ, Saleska SR, Tyson GW, Wrighton KC, Sullivan MB, Rich VI. 2018. Soil Viruses Are Underexplored Players in Ecosystem Carbon Processing. mSystems 3.

7. Nigro OD, Jungbluth SP, Lin H-T, Hsieh C-C, Miranda JA, Schvarcz CR, Rappé MS, Steward GF. 2017. Viruses in the Oceanic Basement. MBio 8:e02129-16.

8. Anantharaman K, Duhaime MB, Breier JA, Wendt KA, Toner BM, Dick GJ. 2014. Sulfur oxidation genes in diverse deep-sea viruses. Science 344:757–760.

9. Anderson RE, Sogin MLML, Baross JAJA. 2014. Evolutionary strategies of viruses and cells in hydrothermal systems revealed through metagenomics. PLoS One 9:e109696.

10. He T, Li H, Zhang X. 2017. Deep-Sea Hydrothermal Vent Viruses Compensate for Microbial Metabolism in Virus-Host Interactions. MBio 8:e00893-17.

11. Labonté JM, Field EK, Lau M, Chivian D, Van Heerden E, Wommack KE, Kieft TL, Onstott TC, Stepanauskas R. 2015. Single cell genomics indicates horizontal gene transfer and viral infections in a deep subsurface Firmicutes population. Front Microbiol 6:349.

12. Williamson SJ, Cary SC, Williamson KE, Helton RR, Bench SR, Winget D, Wommack KE. 2008. Lysogenic virus–host interactions predominate at deep-sea diffuse-flow hydrothermal vents. ISME J 2:1112–1121.

13. Anderson RE, Kouris A, Seward CH, Campbell KM, Whitaker RJ. 2017. Structured populations of Sulfolobus acidocaldarius with susceptibility to mobile genetic elements. Genome Biol Evol 9.

14. Meyer JL, Huber JA. 2014. Strain-level genomic variation in natural populations of Lebetimonas from an erupting deep-sea volcano. ISME J 8:867–80.

15. Coleman ML, Sullivan MB, Martiny AC, Steglich C, Barry K, Delong EF, Chisholm SW. 2006. Genomic islands and the ecology and evolution of Prochlorococcus. Science 311:1768–70.

16. Cuadros-Orellana S, Martin-Cuadrado A-B, Legault B, D’Auria G, Zhaxybayeva O, Papke RT, Rodriguez-Valera F. 2007. Genomic plasticity in prokaryotes: the case of the square haloarchaeon. ISME J 1:235–45.

17. Peña A, Teeling H, Huerta-Cepas J, Santos F, Yarza P, Brito-Echeverría J, Lucio M, Schmitt-Kopplin P, Meseguer I, Schenowitz C, Dossat C, Barbe V, Dopazo J, Rosselló-Mora R, Schüler M, Glöckner FO, Amann R, Gabaldón T, Antón J. 2010. Fine-scale evolution: genomic, phenotypic and ecological differentiation in two coexisting Salinibacter ruber strains. ISME J 4:882–95.

18. Grote J, Cameron Thrash J, Huggett MJ, Landry ZC, Carini P, Giovannoni SJ, Rappe MS. 2012. Streamlining and core genome conservation among highly divergent members of the SAR11 clade. MBio 3:e00252-12.

19. Kashtan N, Roggensack SE, Rodrigue S, Thompson JW, Biller SJ, Coe A, Ding H, Marttinen P, Malmstrom RR, Stocker R, Follows MJ, Stepanauskas R, Chisholm SW, Prochlorococcus W, Kashtan N, Roggensack SE, Rodrigue S, Thompson JW, Biller SJ, Coe A, Ding H, Marttinen P, Malmstrom RR, Stocker R, Follows MJ, Stepanauskas R, Chisholm SW. 2014. Single-cell genomics reveals hundreds of coexisting subpopulations in wild Prochlorococcus. Science 344:416–20.

20. Moulana A, Anderson RE, Fortunato CS, Huber JA. 2020. Selection is a significant driver of gene gain and loss in the pangenome of the bacterial genus Sulfurovum in geographically distinct deep-sea hydrothermal vents. mSystems https://doi.org/10.1128/mSystems.00673-19.
