# Supplementary material for: Diverse Viruses in Deep-Sea Hydrothermal Vent Fluids Have Restricted Dispersal across Ocean Basins
Source: mSystems. 2021 Jun 22;6(3):e00068-21. doi: 10.1128/mSystems.00068-21 (PMC8269205; doi:10.1128/mSystems.00068-21)
Supplement: FIG S5 [file msystems.00068-21-sf005.pdf]

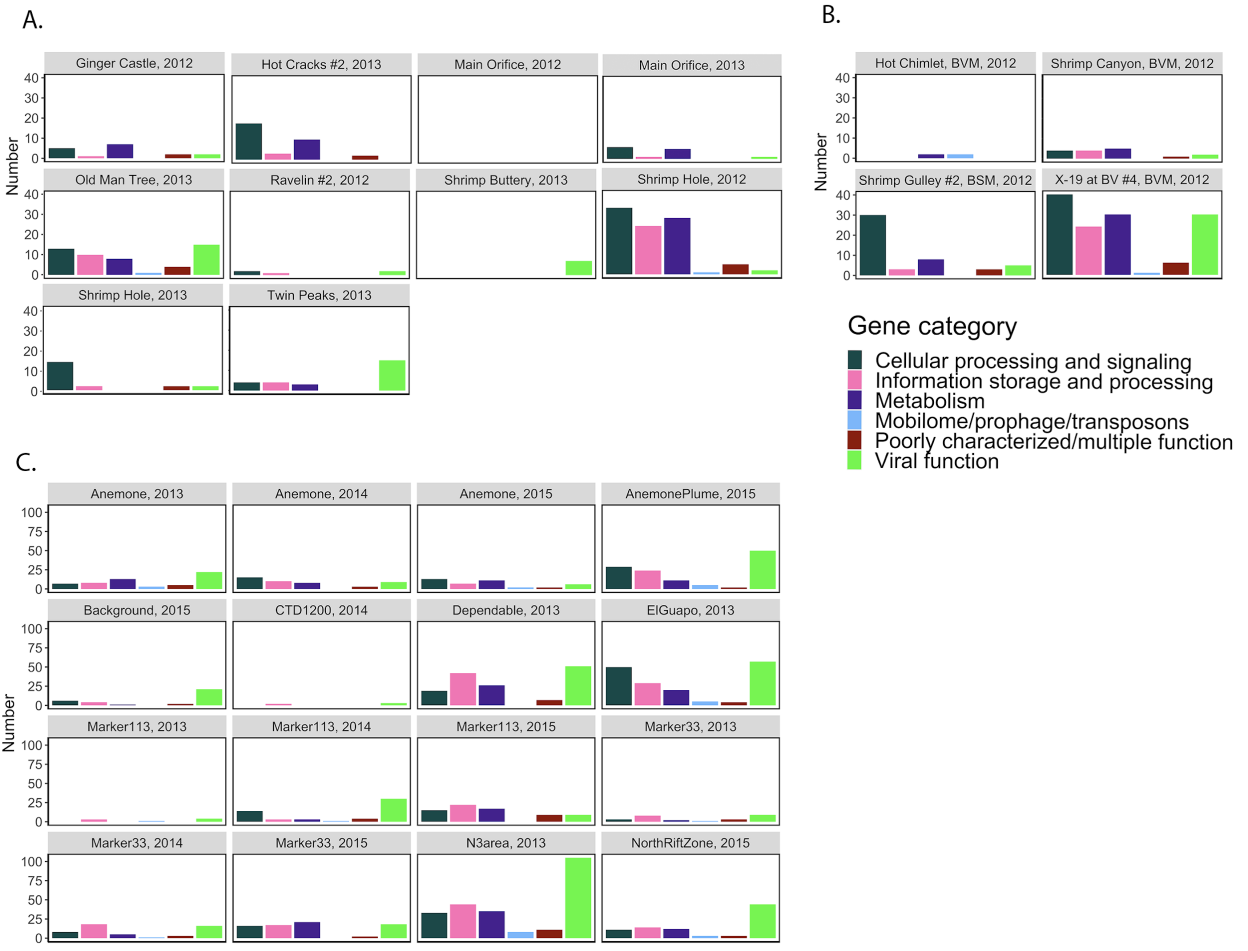
A.
Ginger Castle, 2012
Hot Cracks #2, 2013
Main Orifice, 2012
Main Orifice, 2013
Old Man Tree, 2013
Ravelin #2, 2012
Shrimp Buttery, 2013
Shrimp Hole, 2012
Shrimp Hole, 2013
Twin Peaks, 2013
Number
0
10
20
30
40
B.
Hot Chimlet, BVM, 2012
Shrimp Canyon, BVM, 2012
Shrimp Gulley #2, BSM, 2012
X-19 at BV #4, BVM, 2012
Number
Gene category
Cellular processing and signaling
Information storage and processing
Metabolism
Mobilome/prophage/transposons
Poorly characterized/multiple function
Viral function
C.
Anemone, 2013
Anemone, 2014
Anemone, 2015
AnemonePlume, 2015
Background, 2015
CTD1200, 2014
Dependable, 2013
ElGuapo, 2013
Marker113, 2013
Marker113, 2014
Marker113, 2015
Marker33, 2013
Marker33, 2014
Marker33, 2015
N3area, 2013
NorthRiftZone, 2015
Number
0
25
50
75
100
